# The Arrhythmogenic Face of COVID-19: Brugada ECG Pattern in SARS-CoV-2 Infection

**DOI:** 10.3390/jcdd9040096

**Published:** 2022-03-25

**Authors:** Paul Zimmermann, Felix Aberer, Martin Braun, Harald Sourij, Othmar Moser

**Affiliations:** 1 Department of Sport Science, Division of Exercise Physiology and Metabolism, University of Bayreuth, 95447 Bayreuth, Germany; paul.zimmermann@arcormail.de (P.Z.); othmar.moser@uni-bayreuth.de (O.M.); 2 Department of Cardiology, Klinikum Bamberg, 96049 Bamberg, Germany; martin.braun@sozialstiftung-bamberg.de; 3 Department of Endocrinology and Diabetology, Medical University of Graz, 8036 Graz, Austria; ha.sourij@medunigraz.at

**Keywords:** Brugada syndrome, Brugada ECG pattern, SARS-CoV-2, COVID-19, pandemic

## Abstract

In 1992, Brugada syndrome (BS) was first described; an often unrecognized cardiac conduction disorder mainly associated with unexplained sudden cardiac arrest and consecutive syncope. Nevertheless, the pathomechanism of BS and sudden cardiac death remains mainly explained. Mutations in the cardiac sodium channels, which cause a reduction or functional loss of these channels, are associated with characteristic electrocardiographic (ECG) abnormalities and malignant arrhythmia. The majority of affected people are previously healthy and unaware of their genetic predisposition for BS and might experience ventricular tachyarrhythmias and cardiac arrest potentially triggered by several factors (e.g., alcohol, sodium channel blockers, psychotropic drugs, and fever). Severe acute respiratory syndrome coronavirus 2 (SARS-CoV-2) was firstly identified in Wuhan in early December 2019 and rapidly spread worldwide as coronavirus disease (COVID-19). COVID-19 is typically characterized by a severe inflammatory response, activation of the immune system, and high febrile illness. Due to this condition, symptomatic COVID-19 infection or vaccination might serve as inciting factor for unmasking the Brugada pattern and represents a risk factor for developing proarrhythmic complications. The aim of this narrative review was to detail the association between virus-related issues such as fever, electrolyte disturbance, and inflammatory stress of COVID-19 infection with transient Brugada-like symptoms and ECG-pattern and its susceptibility to proarrhythmogenic episodes.

## 1. Introduction

The severe acute respiratory syndrome associated with coronavirus 2 (SARS-CoV-2) firstly occurred in China in December 2019 and rapidly spread worldwide, ending up as a global pandemic [1]. Although the respiratory system is primarily affected and might cause acute respiratory distress syndrome, coronavirus disease (COVID-19) is associated with cardiac complications, such as infection-associated myocarditis, myocardial injury, or malignant arrhythmias. In this context, inherited or acquired syndromes, i.e., pre-excitation syndromes or Brugada syndrome (BS) might be unmasked, resulting in polymorphic ventricular arrhythmias (PMVT) and sudden cardiac death (ScD) in affected individuals [2].

Up to now, no proven effective therapeutic approach due to COVID-19 treatment, in general, has been established, and the suggestions hereinafter for treatment of COVID-19 patients associated with a Brugada like electrocardiogram (ECG) pattern are derived from different position statements, reviews, and several case reports.

An arrhythmogenic effect of COVID-19 can be expected in patients with an increased risk of cardiac arrhythmias. In this context, sinus tachycardia is a commonly described manifestation in SARS-CoV-2 patients with an overall incidence of 72%, and significant sinus bradycardia is reported in 14.9% of the patients [3]. An increasing incidence of atrial fibrillation (AF) in the context of accompanying COVID-19 infection has been observed and has been reported with a prevalence of 27.5% in COVID-19 patients admitted in intensive care units (ICU) in the United States of America (USA) [4]. Concomitant virus-induced cardiac injury, detected by increased levels of cardiac troponin I, might lead to perimyocarditis or cardiac hypoxemia and may aggravate arrhythmogenicity and increase the risk of paroxysmal AF as well as the risk of non-sustained ventricular tachycardia (nsVT), sustained VT (sVT), and ventricular flutter (VF), as well as PMVT in the setting of QT prolongation [5]—specifically in patients with a history of structural, ischemic, or conduction cardiac disorders. The interaction of the severe inflammatory disease with concomitant disease, such as inherited arrhythmia syndromes, are largely unknown, but interaction might be assumed [6]. The peri-infectious management of COVID-19 patients with ventricular arrhythmias in association with acquired conditions and comorbidities or inherited syndromes remains challenging for potential associated arrhythmogenic risk and individual patient’s therapeutical considerations [6]. Patients with inherited arrhythmia syndromes such as long QT-syndrome, short QT-syndrome, and BS are known for being predisposed to malignant cardiac arrhythmias and ScD, which condition might be enhanced by being exposed to COVID-19 infection [6]. COVID-19 related issues such as stress, fever, electrolyte disturbances, bradycardia, antiviral medication, and other drugs used within emergency and critical care settings might contribute to vulnerability for malignant arrhythmias in such patients [2,6]. In this context, acquired prolongation of QT interval and an incidence rate of 3.6% for life-threatening arrhythmias during hospitalization are reported in patients with no inherited channelopathy. Similarly, this acquired QT interval prolongation may exacerbate the risk of malignant ventricular arrhythmias and associated ScD in patients with inherited long QT syndrome [7]. For patients with known BS and those with revealed Brugada ECG pattern, episodes of lethal arrhythmias may be induced by several COVID-19 associated factors and sufficient management of the acute respiratory failure, hemodynamic and cardiac monitoring is essential to prevent arrhythmogenic complications [8].

Up to now, specific recommendations referring to the detailed care of those patients are lacking and the current suggestions for the most beneficial treatment of those COVID-19 patients are based on existing evidence for ICU management and management of viral infections in general [8]. For these patients with known or suspected Brugada ECG patterns, an aggressive antipyretic therapy and serial daily ECG screenings seem to represent basic tools for patient’s intrahospital management to reduce the risk for syncope and ScD [9,10]. The literature on fever-related arrhythmogenic events in patients with BS or Brugada ECG patterns is currently limited to small series of case reports, but reveals a variable risk of fever-related arrhythmogenic events depending on age, sex, comorbidities, and ethnicity. These factors have to be taken into consideration in the clinical management of these patients also in the context of COVID-19 [11].

The present narrative review—inspired by a clinical case—aimed to describe the arrhythmogenic face of COVID-19 in the context of BS and reveal the potential COVID-19 associated risks for malignant arrhythmias and ScD. We want to focus on some general therapeutical rules and provide recommendations for therapy, management, and monitoring of BS patients during COVID-19 infection.

## 2. Brugada Syndrome

### 2.1. Definition, Clinical Presentation, and ECG Criteria of Brugada Syndrome

The BS, which was firstly described in the early 1990s, was initially characterized by the presence of ventricular arrhythmias and unexplained ScD in otherwise cardiac healthy subjects [12]. The prevalence of BS, estimated to be 2 to 5 in 10,000 inhabitants, seems to be higher in Asian countries than in the western world, and BS usually manifests during adulthood. The average age at the time of ScD is estimated at 41 ± 15 years [13,14].

**Figure 1:**
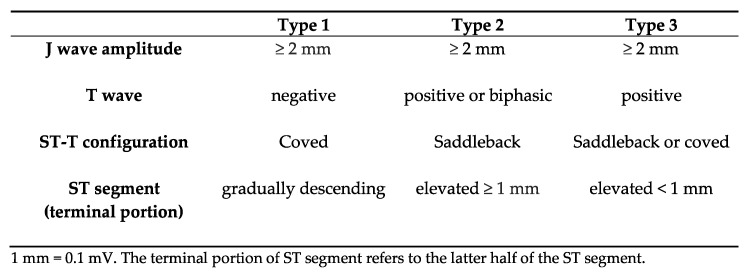
Brugada Syndrome—ECG pattern, inspired by Nishizaki et al., 2013 [16].

The typical ECG presentation is characterized by a coved ST-segment elevation in the right precordial leads V_1–3_ and might be accompanied by a right bundle branch block [15]. This so-called Type 1 ST-segment elevation ECG (Type 1 ECG) is diagnostic for BS and is associated with a high risk of arrhythmogenic events. However, similar ECG changes might be obtained in various normal and abnormal conditions [16]. Type 1 ECG can be unmasked by administration of sodium channel blockers in suspected cases for BS, as well as in symptomatic patients showing a non-Type 1 ECG pattern. This strategy serves as a risk prediction of arrhythmogenic events in patients with transient spontaneous Type 1 ECG or drug-induced ECG alterations [16]. According to the consensus paper from the subgroup of the Heart Rhythm Society (HRS) and European Heart Rhythm Association (EHRA), there are three features of different ECG patterns: Type 1, Type 2, and Type 3 (Figure 1) [13,17].

**Figure 2:**
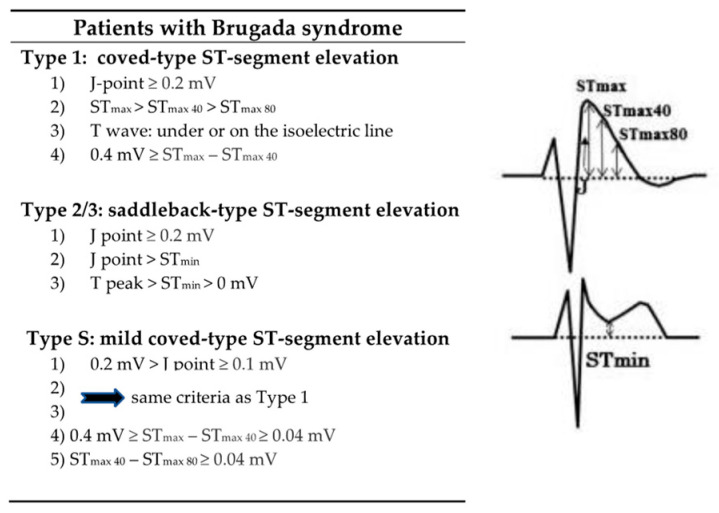
Brugada Syndrome—J-wave amplitude, ST-segment elevation analysis, inspired by Nishizaki et al., 2013 [16].

The typical Type 1 ECG pattern is characteristic for the diagnosis of the BS, but to confirm the final diagnosis, at least one of the following accompanying ECG changes needs to be present: a positive family history of ScD < 45 years of age, a Type 1 ECG in family members, induction of VF or PMVT by programmed stimulation, syncope in history, or documented VF/PMVT. The sodium channel blocker-induced Type 1 ECG, alternatively recorded at a superior lead position, also represents a diagnostic sign for BS based on a consensus report [13,16]. Nevertheless, the diagnosis of BS remains challenging due to the fluctuating nature of ST-elevation over time, which has been recognized as a general feature of this syndrome. Therefore, next to the described Type 1 to Type 3 ECG pattern, the J-point with its characteristic amplitude and configuration has been established as an additional characteristic diagnostic feature (Figure 2) [13,17,18].

Next to the defined ST-segment elevation criteria, depolarization abnormalities resulting in prolongation of QRS- and PR interval or p-wave duration are often detected. A prolongation in the terminal portion of the right precordial S wave, or a prolongated PQ interval (>170 ms), or negative T wave (<105 μV) in V_1_ lead, or fragmented QRS structure are independently associated with life threatening events such as ventricular arrhythmias in patients with BS [19].

### 2.2. Molecular Genetics

BS is a channelopathy with an increased risk of cardiac arrhythmias and ScD, and in this connection, BS is mainly linked to loss-of function mutations in the *SCN5A* gene encoding the alpha subunit of main cardiac natrium channel Na_V_ 1.5, which predominates the cardiac sodium current, I _Na_. Later over, 100 different mutations in the cardiac sodium channel were additionally identified next to the *SCN5A* gene, also in the *SCN10A* gene, which in summary affect nearly 50% of cases [9,13,20,21]. *SCN10A* is regarded as the major susceptible gene for BS, enhancing the opportunity to genotype and risk stratification of patients and family members [20] and to elaborate risk-stratification tools for quantification of ScD risk and target the usage of implantable cardioverter-defibrillator (ICD) [21]. A higher incidence of BS has been reported in consanguineous cases than in sporadic cases and other disorders. Dilatative cardiomyopathy, sick sinus syndrome, AF, or idiopathic VF have been shown to be highly associated with *SCN5A* gene mutations. The electrophysiological abnormalities seem to be determined by the genetic background, and different genotypes of BS may present different clinical phenotypes. In terms of genotype to phenotype correlation, particularly carriers of *SCN5A* gene mutation seem to be predisposed for more pronounced epicardial abnormalities and, therefore, more aggressive clinical presentation [22]. Nevertheless, a negative gene testing does not rule out BS, and the absence of *SCN5A* gene mutation does not correlate with the incidence of ScD [23]. Next to the described *SCN5A* and *SCN10A* gene mutation, mutations encoding the cardiac L-type Ca^2+^ channel alpha subunit, CACNA1c, and the ß-subunit, CACNB2b, as well as alterations in the glycerol-3-phophatedehydrogenase 1-like gene, *GPD1-L* in the sodium channel ß_1_ subunit, *SCN1B,* and in infant death syndrome are considered to be associated with BS and clinical disease [24]. Recently published data revealed a reduced Na_V_ 1.5 current through defective functional Na_V_ 1.5—ß-2-Syntrophin (SNTB2) interaction in native cardiomyocytes of BS patients associated with SNTB2 mutation emphasizing the role of SNTB2 in BS pathophysiology [25]. Furthermore, for heterozygous missense mutation R689H in the *SCN5A* gene—resulting in the loss of protein function—there has been shown some evidence for the coexistence of a Brugada-like ECG pattern and short-QT ECG phenotypes [26]. In this context, El-Battrawy et al. could recently reveal the clinical importance of carrying calcium channel subunit variants in patients with overlapping short QT-syndrome and BS. These patients are typically carrying variants in the L-type calcium channel current (I _Ca-L_) subunits [27].

Due to clinical guidelines, the role of mutation load in BS strongly suggests to perform a gene panel analysis to estimate risk stratification, prevention, and primary prophylactic approach [24].

### 2.3. Mechanisms of Brugada Syndrome and Risk Stratification

During the initial phase of the normal action potential, characterized by inward Na^+^ current and transient outward K^+^ current, disturbances caused by weak Na^+^ current and unopposed outward K^+^ current cause an accentuation of the action potential in the right ventricular (RV) epicardium. Therefore the typical Brugada ECG pattern, i.e., accentuated J-wave and ST-segment elevation, indicate a transmural voltage gradient from the epicardium to the endocardium. Due to the inhomogeneous repolarization in the different areas of RV epicardium and the occurrence of closely coupled extrasystoles, cardiac arrhythmias such as VF or PMVT arise from the RV outflow tract. Electrolyte disturbances, fever, and hormonal influence trigger these extrasystoles with left bundle branch morphology, which might be suitable for successful catheter ablation in the RV in the case of an electrical arrhythmic storm [28].

While the role of programmed electrical stimulation for the risk stratification in BS is a discussed controversy, inducible ventricular arrhythmias on electrophysiological stimulation, spontaneous Type 1 Brugada ECG, and family history on ScD or syncope are strong risk factors for future cardiac events in patients with BS, as reported in a meta-analysis by Wu et al. [29].

### 2.4. Determinants Influencing Brugada-Type ECG Pattern

First of all, it has to be differentiated between the initial reported persistent ST-segment elevation as a characteristic finding in BS and the fluctuating nature of ST elevation as a Brugada ECG pattern, which has been recognized as a general feature of this syndrome over the time [30,31]. The Brugada ECG pattern is variable, dynamic, and often concealed, therefore, multiple factors have been shown to interact with ST-segment alterations [18]. Autonomic imbalances, early repolarization (ER), changes in glucose-induced insulin levels after meals, changes in heart rate, and inflammatory response during elevated body temperature, especially fever episodes, alcohol, and sodium channel blockers, are known as typical risk factors [30,31,32,33,34,35,36]. Among these factors, sodium channel blockers have the most serious potential for adverse cardiac events, but other types and classes of medication are also known for their adverse clinical potential in patients with BS or concealed BS [31,37]. The drugs to be avoided in BS patients are listed at: http://www.brugadadrugs.org, (accessed on 1 March 2022).

### 2.5. Differential Diagnoses of Brugada-Type ECG Pattern

ECG changes that seem to be similar to those in BS can be obtained in various different conditions. Therefore, changes of the cardiac structure, such as left ventricular hypertrophy, arrhythmogenic right ventricular cardiomyopathy (ARVC), or mediastinal tumor compression of RVOT are described as well as electrolyte disturbances like hypercalcemia or hyperkalemia [13,17]. In addition, various central and autonomic nervous abnormalities, ER syndrome, long or short-QT syndrome, as well as acute myocarditis and pericarditis, dissecting aortic syndrome, or acute coronary syndrome have to be taken into consideration in the differential diagnosis of Brugada ECG pattern [16,17,30]. In this context, sodium channel blockers, as ajmaline, have been used to unmask the typical Type 1 ECG pattern. To improve the sensitivity of diagnostic accuracy for unmasking BS, the ECG recording at the upper intercostal lead position of the right precordial leads (V_1–3_) has been established as an additional prognostic tool [13,17].

### 2.6. COVID-19 and Brugada-ECG Pattern

COVID-19 is typically characterized by a severe inflammatory response, high febrile illness, and impairment of the respiratory system and can result in acute respiratory distress syndrome in severe cases. Next to the well-described pulmonary manifestations, COVID-19 may also trigger accompanying cardiac phenomenons [38]. Due to this condition, symptomatic COVID-19 infections might serve as inciting factors for unmasking a Brugada pattern and serve as a risk factor for developing proarrhythmic complications due to virus-related issues such as fever, electrolyte disturbance, and inflammatory stress brought along with COVID-19 infection. These genetic–environmental interactions are of importance for the individual clinical setting in patients with an increased risk for ventricular arrhythmias, either to BS or acquired or fluctuant Brugada-like ECG pattern. Depending on the inherited defect or the acquired syndrome, these patients might be susceptible to proarrhythmogenic effects during various conditions attributed to COVID-19 infection [6], as reported before in BS coinciding with Influenza B and H1N1 Influenza [39,40]. In severely affected and non-severely affected patients with COVID-19, higher prevalence of hypertension, coronary artery disease and derangement of ACE 2 signal pathways, cytokine storm, and myocarditis have not been described, but there is a broad variation of severity in cardiac complications [6,41,42,43], which might trigger arrhythmogenic episodes. High febrile situations are known as a great risk factor for proarrhythmogenic complications and ScD in BS patients [32].

**Figure 3:**
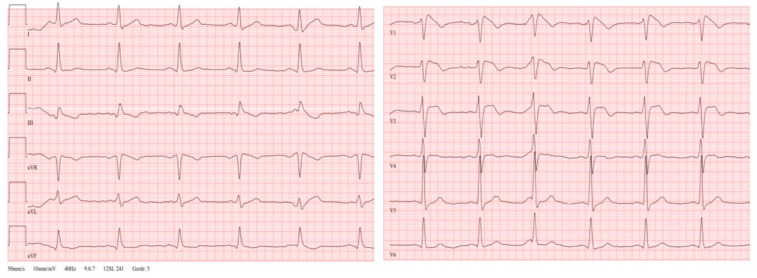
47 year old male and previously healthy patient with transient “Brugada like” ECG pattern in the right precordial leads related to the diagnosis of COVID-19.

**Figure 4:**
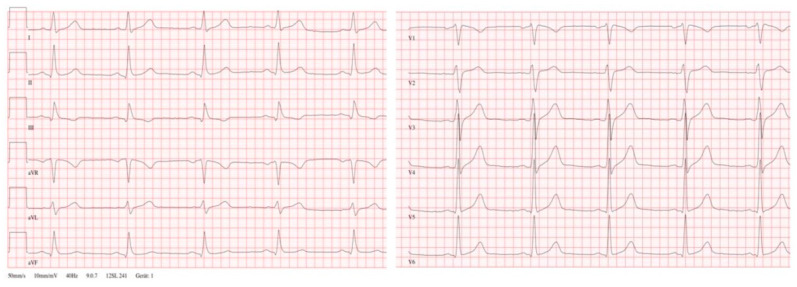
47 year old male and previously healthy patient with transient Brugada like ECG related to the diagnosis of COVID-19 after recovery. By aggressive antipyretic therapy and supportive peri-infectious therapy the patient recovered within 7 days and the fluctuant Brugada like ECG pattern showed gradual recovery and were in the further ECG recordings not detectable.

Referring to this background, a 47-year-old male and previously healthy patient, COVID positive for two days, was admitted to our hospital with shortness of breath and substernal chest pain. On presentation, he was highly febrile (39.5 °C), tachycardic (110 beats/min), and tachypneic (25 breaths/min). His medical history was positive for arterial hypertension and obesity (BMI 32 kg/m^2^). In detail, the patient did not report a history of cardiac abnormalities or previous syncope. The laboratory values revealed an infectious constellation by raised C-reactive protein level (200 mg/L, normal range: 0–10 mg/L) and leukocytosis (14 × 10^9^/L, normal range: 4.0–11.0 × 10^9^/L). The baseline troponin I level at hospital admission was significantly elevated (1.0 µg/L, reference 0.037 µg/L) and decreased during the further observation. The performed chest X-ray did not reveal significant interstitial and airspace opacities related to the diagnosis of COVID-19. During the serial ECG recordings, the patients showed a transient Brugada-like ECG pattern in the right precordial leads (Figure 3). Without any significant clinical cardiac symptoms, the patient was monitored continuously and the bedside echocardiography demonstrated a mildly depressed left ventricular global systolic function with no pericardial effusion or valvular abnormalities. The further clinical presentation and the performed cardiac magnetic resonance imaging revealed a mild course of peri-infectious myocarditis without any notes on cardiac ischemia. By aggressive antipyretic therapy and supportive peri-infectious therapy, the patient recovered within 7 days and the fluctuant Brugada-like ECG pattern was not further detectable (Figure 4).

Acknowledging this clinical case, febrile conditions and inflammatory response as cytokine storm are known as major risk factors in patients with BS in the context of risk stratification for ventricular arrhythmias or PMVT. The sodium channel function is sensitive to temperature—in detail, the temperature may contribute to inactivation and/or decrease of sodium channel expression at higher temperatures [6,10]. Therefore, a minimal functional decline in mutated sodium channels in BS patients or undiagnosed BS patients caused by a minimal higher temperature due to inflammatory response in COVID-19, might translate to a fever-induced arrhythmogenic event due to loss of function in the intact sodium channels [44,45]. Furthermore, the shortening of intraepicardial dispersion of action potential due to elevated temperature might contribute to high-risk conditions for reentrant VT [46]. These potential proarrhythmogenic interactions have been reported in several cases associated with COVID-19 induced fever [9,12,47,48]. The prevalence of Brugada ECG pattern in patients with fever is up to 20 times higher than in fever-free patients, which accentuates the role of fever unmasking Brugada-like ECG pattern and the risk of arrhythmogenic events in BS patients induced by fever episodes [32,49]. Next to the described mechanisms, based on inflammatory response and cytokine storm in high febrile COVID-19 patients, consequent antipyretic therapy is essential for the treatment of these patients, but antipyretic therapy does not serve as an established “unmasking” diagnostic tool. In this context, an interesting case by Adedeji et al. [38] revealed a new-onset Brugada pattern in an afebrile patient with acute COVID-19. In this case, high levels of interleukin 6 (IL-6), procalcitonin, and troponin I support this thesis of maintaining inflammatory setting even in afebrile COVID-19 patients. Close attention should be paid to these circumstances due to transient functional loss of the cardiac sodium channel in even afebrile patients with BS and the associated risk for ventricular arrhythmias and ScD [38].

Next to the mentioned aspects, acidosis and myocarditis have been identified as triggers, unmasking Brugada-like ECG pattern in ICU admitted patients with COVID-19, as reported by Tsimploulis et al. [2]. Bradycardia due to high fever or medication as centrally acting alpha-1 agonist dexmedetomidine or propofol as sedative and analgesic may mediate malignant arrhythmias in BS, as assumed by Gertler et al. and Kloesel et al. [50,51].

Febrile states due to COVID-19 infections as an environmental–genetic interaction may unmask certain BS patients. Adler et al. could reveal a prevalence of Brugada Type 1 ECG pattern in 2% of the 402 included COVID-19 patients with fever [49]. The detection of asymptomatic BS during COVID-19 disease remains challenging, as reported in several case reports, because of the variable patient’s clinical presentation and the often underdiagnosed ECG profiles [9,52]. Nevertheless, the threshold to record an ECG at admission and repeat frequent recordings during the follow-up in patients with BS or a Brugada-like ECG pattern should be low to minimize the potential risk of COVID-19 related proarrhythmogenic complications [53]. These recommendations are also valid for the management of arrhythmia syndromes in children, whereas fever-triggered malignant ventricular arrhythmias represent the major concern in the context of COVID-19 associated with BS and concealed BS, as stated by the Association for European Paediatric and Congenital Cardiology (AEPC) position paper [54].

As mentioned before, a Brugada-like ECG pattern is unmasked in febrile states, but there might arise small evidence that even in the absence of fever where the viral infection per se may be responsible for the typical changes in the ECG by direct cardiotoxicity [55].

## 3. General Recommendations for Therapy, Management, and Monitoring of Brugada Patients during the COVID-19 Pandemic

Big challenges remain in the clinical management of patients with complex cardiac syndromes, such as BS or transient Brugada like ECG pattern in patients with COVID-19 because, to date, current recommendations for the optimal treatment of those patients have been based on previous evidence for intensive care management published prior to the existence of COVID-19 [8].

Many questions on ICU management of BS patients remain difficult to be clarified, including the estimation of arrhythmogenic risk, thromboembolic complications due to heart rhythm abnormalities or vasculitis, inflammation associated myocardial dysfunction, the role of invasive and noninvasive ventilation, and sedative and analgesic medication on ICU [8].

**Figure 5:**
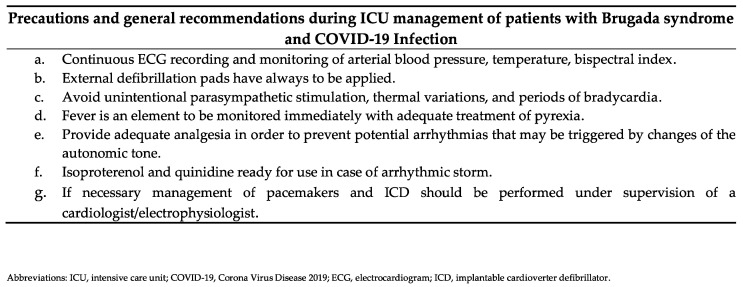
Precautions and general recommendations during ICU management of patients with BS and COVID-19 Infection, modified from Dendramis and Brugada, 2020 [8].

In addition to the known respiratory problems in cardiac-affected COVID-19 patients, they may develop cardiac complications such as myocarditis, heart failure, and a high-risk condition for ventricular arrhythmias. For this reason, patients with structural or primarily arrhythmogenic heart disease have a greater risk for complications, and any factors that unbalance the parasympathetic or sympathetic traffic have to be avoided. Due to the temperature-sensibility of the mutated sodium channels, the general management and monitoring of these patients should be based on special vigilance of continuous ECG monitoring, fluid management, temperature, and blood pressure measurement, as well as adequate treatment of pyrexia (Figure 5).

Isoproterenol infusion or combined application of quinidine as an effective acute treatment in patients with BS with an electrical storm of VF has been evaluated in several case reports and reviews and is recommended as first choice therapy in the management of BS patients with pacemakers or implantable cardioverter defibrillator (ICD) [8,56,57,58].

The drug management on ICU in BS patients with high arrhythmogenic risk has to be considered very carefully by a cardiologist, and drugs with potential arrhythmogenic potential should be avoided. Propofol, commonly used as an induction agent for sedation in ICU, may affect significant ST-segment alterations in known or concealed propofol infusion syndrome (PRIS), characterized mainly by metabolic acidosis, cardiac dysfunction, rhabdomyolysis, or renal failure in patients undergoing long term treatment. In the context of this rare complication, widening of the QRS complex, ventricular arrhythmias, asystole, and Brugada Type 1 ECG pattern have been described. Up to date, clinical experience does not recommend avoiding the short-duration application of propofol in general, but extreme caution is advised in the long-term application during ICU treatment [8,56,59].

Benzodiazepines, narcotics, and opioids have not been reported for such adverse events. Alpha receptor agonists, such as norepinephrine and beta-receptor antagonists, might worsen ECG parameters and unmask a Brugada pattern in cardiac affected BS patients, whereas alpha-receptor antagonists and beta-receptor agonists are reported for ECG improvements and decrease of ST-segment elevations [8]. An increased vagal tone might contribute to increased arrhythmogenic vulnerability, so that the anticholinergic action of applicated atropine and scopolamine in acute therapy could positively influence the ST segment alterations in the context of Brugada and COVID-19 [56]. Medication such as chloroquine, as an antimalarial drug with relation to quinidine, or hydroxychloroquine and azithromycin for treatment of COVID-19 patients, have a known association with QTc prolongation. Therefore careful attention to drug interactions due to susceptible proarrhythmogenic risk in patients with inherited arrhythmia syndromes as BS is recommended [60,61,62,63].

**Figure 6:**
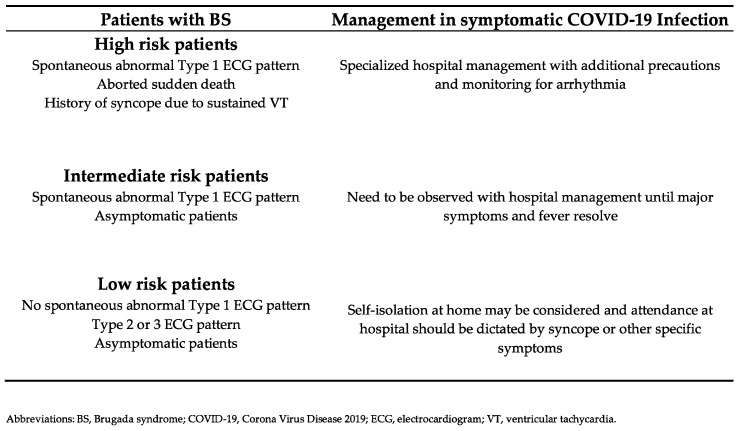
BS and symptomatic COVID-19 infection. Risk stratification and flow charts for hospital or home care management, based on Dendramis et al., 2020 [8].

In the high-risk population of BS patients with spontaneous Type 1 ECG pattern, or history of syncope, or sVT, and accompanying symptomatic high febrile COVID-19 infection, a hospital admission with special precautions and monitoring is necessary independently from the presence of COVID-19 typical symptoms (e.g., hypoxemia) or pulmonary manifestations. Patients with asymptomatic spontaneous Type 1 Brugada pattern, defined as intermediate risk, should also be admitted to hospital treatment until fever and severe symptoms refine. Patients classified as low-risk patients, defined as drug-induced Type 1 Brugada pattern, as well as those without spontaneous Type 1 Brugada pattern even during fever, are recommended for resting and self-isolation at home, aggressive fever reduction, preferentially with paracetamol and should attend to hospital after the occurrence of syncope or specific COVID-19 related symptoms (Figure 6) [6,8].

Another aspect that arises in the context of inherited arrhythmogenic patients and the COVID-19 pandemic is the application of COVID-19 vaccines in these patients. The vaccines approved by the European Medicines Agency (EMA) and currently most widely used are based on two different mechanisms: mRNA (Comirnaty—BioNTech/Pfizer, COVID-19 Vaccine Moderna) and adenoviral vectored (COVID-19 Vaccine Janssen, Vaxzevria—AstraZeneca). All of them are known for rare side effects. In this context, fever episodes were reported by 11 and 16% in older and younger mRNA Comirnaty vaccine recipients, respectively, after the second dose [64,65]. Therefore, a hospital vaccination center management is discussed for these patients in order to prevent arrhythmogenic risk and guarantee greater safety [66]. In BS patients with low and intermediate arrhythmogenic risk, fever prophylaxis prior to vaccination and close-meshed fever monitoring is recommended, whereby in BS patients with high arrhythmic risk for ventricular arrhythmias or spontaneous Type 1 ECG with a history of arrhythmic syncope, hospitalization may be considered even without fever, especially in patients with absence of implanted defibrillators. In this context, Kokawa et al. reported an interesting case of a 41-year-old male BS patient who was previously equipped with a subcutaneous implantable cardioverter defibrillator (S-ICD) because of a VF episode in the past. After the second dose of mRNA COVID-19 vaccination (Comirnaty), he developed high febrile episodes and a transient BS ECG pattern with consequent appropriate S-ICD shocking. Adverse reactions, such as high febrile episodes and pyrexia, are frequently observed associated with the second dose of COVID-19 vaccination. This case impressively shows the medical necessity of accompanying antipyretic therapy pre-COVID-19 vaccination in high-risk BS patients [67]. Okawa et al. demonstrated a case of unmasking a Type 1 ECG pattern in a BS patient several days after COVID-19 vaccination without fever, which highlights the importance of ECG screening prior to COVID-19 vaccination to detect latent BS patients and to reduce the risk of ScD and malignant ventricular arrhythmias in association with the COVID-19 vaccination [68]. However, a case-by-case evaluation in these high-risk patients should be performed considering the patient’s inflammatory response to vaccination, age, and previous vaccination response history [66,67,68].

In a comprehensive review on BS unmasked by fever in general, sixty articles were reviewed and 3% of the analyzed 71 patients had a persistent Brugada ECG pattern, whereas 86% of the patients recovered completely after adequate multimodal treatment of anti-inflammatory and antipyretic treatment. One interesting characteristic that was evaluated in these reported data was if there was any correlation of better outcomes with ICD placement. Based on these findings, the Brugada pattern has favorable outcomes if the underlying conditions are treated adequately. These facts confirm the preferential conservative treatment and the patients with Brugada pattern unmasked by fever do not need long-term ICD placement or antiarrhythmogenic medication [69]. Current literature is limited by the lack of reliable and detailed data on COVID-19, BS and related arrhythmias. Cardiac arrhythmias, as life-threatening ventricular arrhythmias in the context of COVID-19 as an inflammatory disease and Brugada ECG pattern, have to be investigated in further clinical studies to detect the exact arrhythmogenic mechanisms involved and to recommend prophylactic measures and safe therapies for in-hospital and out-of-hospital management. As long as no data is available, temporary use of a wearable external cardioverter defibrillator life vest seems to represent an effective alternative in patient’s vulnerable condition, estimated by the cardiologist in charge.

The main strength of our descriptive narrative review is the limited literature in this scientific area and our reporting might pave the road to further scientific effort in this field of BS and accompanying COVID-19. It highlights the importance of a multidisciplinary approach to improve BS in the patient’s in-hospital and out-of-hospital management.

## 4. Conclusions

Specific data and recommendations on treatment, management, and monitoring of patients with increased baseline risk due to previous cardiac comorbidities, such as a Brugada Type 1 ECG pattern or BS in concomitant COVID-19 infection, are lacking. Special vigilance avoiding factors and situations that are known for high potential arrhythmogenic risk is strongly recommended. Therefore, fever should be treated fast and adequately, other alternative fever-causing etiologies should be ruled out, and bradycardia and electrolyte disturbances should be avoided. During the hospital treatment or ICU management, the intrinsic arrhythmogenic risk should be reduced by increased awareness of possible drug and medication interactions and intensive ECG monitoring. A baseline ECG should be obtained for all COVID-19 patients; those with a known or suspected Brugada ECG pattern should be managed with aggressive antipyretic therapy and receive daily serial ECG readings to detect potential asymptomatic arrhythmogenic episodes. In the clinical setting of high-risk COVID-19 patients with BS, attributed to a genetic–environmental interaction, these patients should be admitted to hospital treatment for continuous ECG monitoring and symptomatic therapy.

## Data Availability

Not applicable.
